# The truth project paper one—how did victims and survivors experience participation? Addressing epistemic relational inequality in the field of child sexual abuse

**DOI:** 10.3389/fpsyt.2023.1128451

**Published:** 2023-06-02

**Authors:** Claire Barker, Stephanie Ford, Rebekah Eglinton, Sally Quail, Daniel Taggart

**Affiliations:** ^1^IICSA, London, United Kingdom; ^2^School of Health and Social Care, University of Essex, Colchester, United Kingdom

**Keywords:** institutional child abuse inquiry, child sexual abuse, trauma informed approach, epistemic justice, testimonial sensibility

## Abstract

The last 30 years has seen an exponential increase in Historical Institutional Abuse Inquiries.^1^ One feature of these has been to place adult survivor voices at the center of Inquiry work, meaning that child abuse victims and survivors^2^ are engaging with Inquiries, sharing their experiences, with this participation often presented as empowering and healing. This initiative challenges long held beliefs that child sexual abuse survivors are unreliable witnesses, which has led to epistemic injustice and a hermeneutical lacunae in survivor testimony. However to date there has been limited research on what survivors say about their experiences of participation. The Truth Project was one area of work of the Independent Inquiry into Child Sexual Abuse^3^ in England and Wales. It invited survivors of Child Sexual Abuse to share their experiences including the impacts of abuse and their recommendations for change. The Truth Project concluded in 2021 and heard from more than 6,000 victims of child sexual abuse. The evaluation of the Trauma Informed Approach designed to support survivors through their engagement with the project was a mixed methods, two phase methodology. A total of 66 survey responses were received. Follow-up interviews were conducted with seven survey respondents. The Trauma Informed Approach was found to be predominantly helpful in attending to victim needs and minimizing harm. However, a small number of participants reported harmful effects post-session. The positive impacts reported about taking part in the Truth Project as a one-off engagement challenges beliefs that survivors of child sexual abuse cannot safely talk about their experiences. It also provides evidence of the central role survivors should have in designing services for trauma victims. This study contributes to the epistemic justice literature which emphasizes the central role of relational ethics in the politics of knowing, and the importance of developing a testimonial sensibility when listening to marginalized groups.

## Introduction

### Prevalence and impacts of CSA

Definitions of Child Sexual Abuse (CSA) vary but most refer to “forcing or enticing a child to take part in sexual activities” (1). Prevalence rates vary; estimates globally suggest somewhere between 8–31% of girls and 3–17% of boys are victims of CSA (2). In England and Wales where this study was conducted, it is estimated that 3.1 million adults experienced CSA before the age of 16, equivalent to 7.5% of the population (1).

The long-term health impacts of CSA are significant (3). These include increased exposure to a range of mental health conditions; depression (4), anxiety disorders (5), psychosis (6), dissociative disorders (7), in addition to Complex Post-traumatic Stress Disorder (C-PTSD) and Post-traumatic Stress Disorder (PTSD) (8). There is also increased risk of chronic physical health conditions, including respiratory conditions (9), diabetes (10), and premature death (11).

### Historical institutional abuse Inquiries

Over the past three decades, alongside growing awareness of the impacts of child abuse, there has been increased recognition of public institutions involvement in its perpetration and cover up (12). Increased awareness has arisen from media exposure and campaigning activism by victims and survivors (13). Over the past two decades there have been Historical institutional abuse (HIA) Inquiries conducted in all four countries of the UK, Australia, Canada, New Zealand, Ireland, the Netherlands, Sweden, Switzerland, Norway, Jersey, and Germany (12).

HIA Inquiries differ in focus, structure and remit; with some focusing on the failings of one institution, such as the Ryan Commission into Catholic Church administered residential care in Ireland, while others including the Australian Royal Commission have looked into the conduct of a broader range of institutions (14). However, one emergent common feature has been the shift in emphasis toward the centering of survivor perspectives, a shift that has been described as the “turn to testimony” (15). This “therapeutic turn” led to HIA Inquiries developing new forms of public engagement that augmented the traditional method of providing evidential testimony under oath at a Public Hearing.

However, while HIA Inquiries claim to have become survivor centered and their participation has been framed in therapeutic justice terms (16), there is limited evidence detailing what survivor participants report about their experience of engagement. One study, conducted following the Northern Ireland HIA Inquiry, highlighted the use of therapeutic discourse around healing and closure, and linked it to Transitional Justice frameworks focused on recognition and reparation for historical harms (17). The study found that there was mixed evidence about how victim centered their participation was, more than half said it was a positive experience, while a sizable minority found it to be exposing (39%) and led to longer term emotional consequences (29%). Almost half the participants described the experience as traumatizing (47%). The study concluded that participation should not focus exclusively on therapeutic outcomes for victims and survivors, but to take a broader perspective on victim needs. A further study looked at the experiences of participants in the Australian Royal Commission into Institutional Responses to CSA (18). The research included interviews with 26 survivors, with a majority reporting respectful and humane treatment, that contrasted with their previous experiences of minimization and denial by institutions. The recognition of participants by the Commission was argued to bestow a “dignification” that can counteract the traumatic shame of CSA, and positions Inquiries as public spaces in which “therapeutic politics” (19) can promote both personal healing and social change.

### Epistemic injustice and the politics of knowing

One conceptual framework that can be used to understand the historic marginalization of CSA survivor testimony, and the shift toward survivor centered processes in HIA Inquiries is Miranda Fricker’s work on epistemic injustice (20). Epistemic injustice refers to a person being “wronged specifically in their capacity as a knower” (21). Fricker’s theory is specifically concerned with the ways in which the reliability of testimony is predicated upon the listener’s evaluation of the credibility of the speaker. The credibility of the speaker is a key issue when the listener is drawing conclusions, based on the identity markers associated with the speaker as well as what they are trying to communicate (22). For example, in mental health, people with psychiatric diagnoses may be seen as less reliable because of long standing societal discourses about madness and unreason, making their testimony suspect by virtue of their association with this group (23). People who have experienced CSA have a long history of being described as unreliable witnesses to their own experiences, with institutional, disciplinary, and scientific power being deployed to undermine their testimony (24).

Examples of CSA survivor testimony being disregarded as unreliable can be found within the practices of the psy disciplines of psychology and psychiatry. False Memory Syndrome (FMS) is a psychological theory originally developed through experimental studies examining the capacity of research participants to be manipulated into believing fictitious things had happened to them, thereby creating “false memories.” Very quickly FMS theory was assimilated into criminal cases where it was used by the defense to argue that the complainant had been manipulated into believing she was abused by counsellors and therapists fixated on finding evidence of childhood trauma to explain psychological and psychosomatic complaints in adulthood (25). The development of a discourse suggesting some CSA survivors were falsely remembering sexual abuse had a profound impact on the public imagination and was picked on by the media as evidence of a moral panic around CSA (26). This “discourse of disbelief” (27) has been mobilized to undermine the testimony of survivors not only in family cases, but also in institutional settings, and has been characterized as a discursive contest about knowledge and pitting scientific evidence against survivor testimony (19). From an epistemic injustice perspective, the epistemic credibility of CSA survivor testimony has been undermined by claims to science of FMS psychologists. This has led to a hermeneutical lacunae where survivor knowledge have been superseded by widespread skepticism about the veracity of the extent of CSA in contemporary society (19). This knowledge gap creates a hermeneutical injustice (21) for survivors who lack a shared, socially sanctioned framework to discuss their experiences of CSA, leading to limited opportunities to disclose, seek help, and a lack of different forms of justice. The establishment of IICSA and other HIA Inquiries, therefore offer an opportunity to provide survivors with a form of testimonial justice, while also creating discourses that validate survivor identity and enable CSA to become an articulated, shared and destigmatized social concern (12).

Why this matters so much can be understood in relation to Fricker’s definition of social power; “a practically, socially situated capacity to control others’ actions, where this capacity may be exercised (actively or passively) by particular social agents, or alternatively, it may operate purely structurally” [(21), p. 14]. In this instance, survivors of CSA are subject to control over the believability of their testimony about abuse, with this social power being exercised indirectly by social agents in the form of scientific psychology, and research into false memories. However, while the capacity to control credibility is passive and indirect, psychologists are not necessarily directly intervening to question the testimony of an individual survivor, although they might be, the consequences are active and structural. In other words, by creating doubt about the testimony of non-recent CSA survivors in general, there are practical consequences for specific survivors when they try to access justice or disclose to other forms of authority about their non-recent abuse. The setting where we can see this structural form of social power play out most forcefully is the criminal justice system, where evidence about false memory and unreliable testimony is used routinely by defense barristers to cast doubt on the credibility of survivors, and in some cases this may include expert witness psychologists providing testimony to the research evidence and how this is relevant in a particular case (28). There are significant gaps between prevalence of CSA and reporting, prosecution and conviction rates; with estimates of around 500,000 children being abused in a single year in England and Wales, compared to police reports of 67,675 in 2021 cases, and 3,420 convictions for CSA related offences the previous year (29). Given this gap between estimated prevalence and conviction rates, it seems improbable, to say the least, that the over-reporting of non-recent CSA is a greater risk than under-reporting, and yet a wealth of social power, from psychological science, through expert witness testimony in courtrooms, is deployed to undermine survivor testimony. It is therefore crucial that Public Inquiries like Independent Inquiry into Child Sexual Abuse (IICSA), which operates a different form of social power and one that validates survivor testimony, are examined with an epistemic justice lens, to understand how they can offer restorative justice to a previously maligned and stigmatized group.

### Research aims

The current HIA literature suggests that Inquiries increasingly aim to be survivor centered, that sharing their experience can have therapeutic outcomes, and that there is limited and mixed evidence regarding how well they attend to victim needs. Two questions arising from this are, how should HIA Inquiries address survivor needs and avoid causing harm? More specifically, are Trauma Informed Approaches (TIA) an effective model for engaging survivors in Inquiries and if so, in what ways?

A final research aim concerns analyzing the work of HIA Inquiries through an epistemic injustice lens. This allows concepts from the epistemic injustice literature to be operationalized in practical HIA Inquiry settings, and can enable insights into how elements of epistemic justice can be linked to the testimonial justice and trauma informed care that are offered at HIA Inquiries.

This study aims to address these questions by drawing on data generated from victims and survivors who participated in the Truth Project. A brief overview of the Truth Project, and the TIA literature will be presented to situate the findings in context.

### Independent inquiry into child sexual abuse and the truth project

IICSA was established as an Inquiry in 2015 to investigate “whether public bodies and other non-state institutions have taken seriously their responsibility to protect children from sexual abuse in England and Wales, and to make meaningful recommendations for change, to ensure that children now and in the future are better protected from sexual abuse” (3). The Truth Project was a core part of IICSA, linked to one of the Inquiry’s terms of reference from the UK Home Secretary, to “consider the experiences of survivors of child sexual abuse, providing opportunities to them to bear witness to the Inquiry, having regard to the need to appropriate support in doing so” (30). The Truth Project was accountable to the Inquiry Chair and Panel, and a Restriction Order was put in place to ensure the anonymity of participants. There was a statutory obligation to report all allegations of child abuse to the police (31).

The Truth Project was piloted in 2015 and from 2016 to 2021, over 6,000 adult victims and survivors shared their experiences via face-to-face sessions, telephone and video calls, or in writing. It was co-designed with the IICSA’s Victims and Survivors Consultative Panel (VSCP), a group of CSA survivors who have expertise in the field. The experiences shared with the Truth Project were used for research to ensure survivors’ voices were included, to add to the evidence in the field of CSA, to help IICSA in its development of recommendations to prevent CSA in the future and improve institutional responses (32).

### Trauma informed approaches

TIAs are an organizational level intervention that recognize the health and social impacts of traumatic stress and have an awareness of the ways that institutions may reenact traumatic dynamics when delivering services to victims and survivors (33). TIAs recognize the impacts of trauma, while also structuring the organization and the practices of staff to minimize the risks of retraumatization.

TIAs have been applied in a range of settings, including: child welfare units (34), psychiatric inpatient units (35) justice systems (36) domestic violence shelters (37), and homeless services (38).

The evidence base for TIA implementation and impact is mixed. A recent systematic review of 32 TIA studies across a range of service settings for various typologies of abuse, found a significant reduction of PTSD symptoms in around half the studies examined (11 of 23) (39). A review of TIAs impact on child welfare settings, found implementation variability, with staff training being the most frequently evaluated form of intervention (40). In community adult mental health and addiction settings, the closest evidence base to the area investigated in this study, there is limited evidence available. Studies show some reduction in PTSD symptoms, improved service engagement and reduced use of emergency care, but no impact on other outcomes such as substance misuse (41–43).

### Independent inquiry into child sexual abuse’s model of trauma informed approaches

IICSA developed a TIA model for the Truth Project (TP) that was designed by psychology staff and members of the VSCP. VSCP members did an end-to-end walk through of the model to evaluate what it would be like for victims to participate, making adjustments to environmental and interpersonal features accordingly. For example, VSCP members booked on to a session to check the booking process offered choice and control. They attended and participated in a Truth session to evaluate the staff skills and environmental considerations. VSCP members had a background in sexual violence services and so imported this knowledge, service philosophy, and therapeutic orientation; as well as offering lived experience. The TIA model was therefore a hybrid drawing on the literature but also survivor expertise. It emphasized five components: (1) Recognizing that the experience of child sexual abuse is subjective and individuals should be respected; (2) Being aware that trust is not to be taken for granted, but fostered; (3) Empowering victims and survivors in their interactions with the Inquiry; (4) Prioritizing the safety and well-being of victims and survivors and working to prevent retraumatization; (5) Acknowledging the impact of child sexual abuse and institutional failures, therefore, looking out for staff wellbeing. The TIA was operationalized in a range of ways through staff training, and integration into all Truth Project processes such as communications policies, complaints processes, as well as building and website design. This was supported by a full-time consultation service delivered by clinical staff. Staff support and training have also been evaluated as part of this study and are described in another paper in this issue (Barker, Taggart, Gonzalez, Quail, Eglinton, Ford, and Tantam).

There was also a three-stage trauma model support service available to all Truth Project participants, delivered by a team of counsellors and support workers, and co-designed by the VSCP. Participants could opt-in to the service at any time, which a majority did (78%*), and utilize as much as they chose to. This included a support worker offering telephone-based support prior to the Truth session to plan around support needs, identifying any risks and session preferences; emotional support on the day of the session, and follow-up support after the session for up to 2–3 weeks.

## Method

### Participants

Ethical approval for the study was sought via consultation with IICSAs independent ethics research panel. People were eligible to participate in the study if they had attended a TP session. Eligible participants were identified through the Inquiry’s Victims and Survivors Forum (VSF), a platform IICSA established to engage with victims and survivors in order to consult them on specific projects. A total of 66 individuals completed the mixed methods survey. The demographics of this group, shown in Table 1 below, differed when compared to the general population of Truth Project participants.^4^

**Table 1:** Participant demographics.

Age		Gender		Ethnicity	
Over 65	9	Female	53	White/British	52
56–65	22	Male	11	Jewish	1
46–55	16	Non-binary	1	Romany/Trinidadian	1
36–45	11	No response	1	Gypsy/traveler	1
26–35	5			Black British	1
Under 25	3			British Indian	1
				Not answered/other	9

Twelve participants were contacted for a follow up semi-structured interview. They were recruited using a purposive sampling strategy, identifying individuals who appeared to be able to offer further rich data, based upon what they had already shared. Further, individuals were selected to reflect the diversity of the sample; including ethnicity, gender, age and time since Truth Project session (44). Seven participants responded and completed the telephone interview. Of these two were male and the remaining five were female, indicating a higher proportion of male participants than was seen in the overall sample. However, the majority of those who responded were white British and, as such, ethnic diversity was not reflected.

Of the 66 participants who completed this survey, the majority took part in the TP in person (50 people). Of the remaining participants, 10 took part over the phone, three over video link and three in writing. The majority had taken part in the Truth Project 1–3 years ago, with 15 people having participated more than 3 years ago, and eight having done so within the last year. Of this sample, 36 took up the support offer, while 30 did not.

### Procedure

A mixed-methods survey was developed based on IICSA’s model of TIA by two researchers who were not involved in the setup of the model but were IICSA staff (CB & SF). Participants were asked to rate the extent to which the Truth Project (1) Enabled them to feel empowered in their engagement (2) Treated them as an individual (3) Acted in a trustworthy way (4) Avoided retraumatization and (5) Created a safe environment for them in their engagement. They were asked five closed questions based on the extent to which the Inquiry fulfilled each of the five TIA principles, with a range from 1-not at all to 5-all of the time. In addition to ratings, participants were asked to provide details of instances where they felt the Truth Project either did or did not fulfill these aims. A further open question was asked about their overall view of how the TIA was implemented and for any other aspects of their experience with the Truth Project they wished to report.

A purposive sampling (44) strategy was used to identify participants for follow-up interviews. The semi-structured interview allowed for elaboration on aspects of participants’ responses to the initial survey, in particular any issues raised by participants suggesting a negative experience.

### Analysis

The qualitative data was entered into Microsoft Excel for thematic analysis. The semi-structured interviews were recorded, transcribed and analyzed, and then added to the survey data to produce one qualitative dataset. The amalgamation of different forms of qualitative data garnered from survey and semi-structured interview respectively, was done for pragmatic reasons due to the Truth Project’s fixed lifespan and time limits on the study. However, attention was paid during analysis to how the semi-structured interview data could complement the larger number of survey responses, without overwhelming it.

Two authors (CB and SF) conducted the qualitative analysis, with another (DT) cross checking coding decisions to ensure reliability. Research supervision meetings were held throughout analysis to discuss emergent themes and to manage differences in coding. The qualitative data from both survey and interviews were analyzed using a six stage Thematic Analysis. The first stage required a thorough familiarization with the data, followed by a systematic identifying and labeling to group the data relevant to the research question. Key patterns or themes were identified followed by a review of those themes. The final two stages entailed a defining and naming of themes, leading to the final weaving together of the themes and narrative to provide the analytic conclusions (45). Given that some of the open-ended questions were generated based on the TIA model (trustworthiness, safety, retraumatization, empowerment and individual care), the data was not analyzed in a purely inductive way, but the data was still subjected to line by line coding to generate initial codes, before developing and refining themes across the dataset, and finally combining and defining themes. Rigor and trustworthiness were addressed in the analysis through the development of a reflexive audit trail of decision making, research supervision, use of data in the findings to promote confirmability, and ensuring credibility through engagement with the full data set before developing themes (46). The VSCP were also consulted throughout the analysis to check on their contributions to the model’s development.

## Findings

The findings are divided into eight themes. The six TIA related themes: overall experience of the TIA; retraumatization; individual recognition; trustworthiness; empowerment, choice and control; and safety, were all asked about directly in the survey. The other two themes; being believed, the long-term consequences and need for support, contain qualitative data that was not asked about but which emerged as themes during analysis. Quotes are attributed to pseudonyms to ease cross referencing and protect anonymity.

### Overall experience of the trauma informed approaches

Five (7.6%) of participants indicated that a TIA is important when working with victims in an Inquiry setting. Of those who spoke about the approach, responses were positive:

*“…it’s just not going to work unless you are trauma-informed. It’s a framework that’s been very well constructed …” Thomas (interview).*

Many reflected upon the processes and approaches they found helpful. This varied from identifying behaviors used by Inquiry staff, such as listening and showing respect, to environmental considerations:

*“Apart from the staff being obviously well trained it was the little acts of care like making water, drinks and tissues available. Making the offices quiet, comfortable, calm and private - that made a big difference to me and helped a lot.” Kelly (survey response).*

### Feeling retraumatized

When asked whether participants felt that their engagement with the Truth Project caused them to re-experience trauma, 36.3% (*n* = 24) reported that they did not feel traumatized at all or very little. However 39.4% (*n* = 26) reported that they did feel somewhat traumatized or felt traumatized most of the time, while 24.2% (*n* = 16) did not indicate either way.

In general, 9.1% (*n* = 6) participants reported that the nature of discussing their experience of CSA is, in itself, traumatizing. However they did not attribute this to having been heightened due to the approach taken by the Truth Project:

*“I did not feel that anything the project did re-traumatized me. Just the act of talking about it all, some bits for the first time was, of course, very traumatic but this was not contributed to by the project.” Kelly, (survey response).*

Others reported there was comfort in knowing what the Truth Project was about and what to expect. This prepared them ahead of their session and helped to prevent them from being re-traumatized as their experience was, somewhat, predictable:

*“I didn't feel traumatized because it was what was written on the tin was inside.” Barbara, (interview).*

Some participants questioned whether a degree of re-experiencing can be seen as part of the healing process:

*“In some respects I think it was helpful to have had some degree of re-experiencing what had happened, because with distance and maturity I was more able to label and acknowledge what those feelings were, whereas previously I would not let myself go anywhere near them for fear of being overwhelmed.” Amelia, (survey response).*

However, 10.6% (*n* = 7) found the process retraumatizing and 3% (*n* = 2) reported the after-effects as long-lasting. The consequences of being retraumatized were generally described in terms of ongoing mental health symptoms:

*“I suffered a period of depression, PTSD and anxiety having been triggered as a result of sharing my experience with the Truth Project. This has lasted a number of years.” Rachel (survey response).*

More commonly, responses from participants indicated that the majority still found benefit in having shared, despite the consequences for their mental health:

*“For a few weeks after being at the project I had flashbacks, bad dreams and a lot of unknown fears, but I have to say it was worth every ounce of pain.” Simon, (survey response).*

### Being recognized as an individual

When exploring the extent to which their individual experience of CSA was recognized when engaging with the Truth Project, 84.8% (*n* = 56) felt the Inquiry acknowledged their individual experience of CSA and 6% (*n* = 4) felt that their individual experience was not acknowledged very much, while 9.1% (*n* = 6) did not indicate either way.

Some said they appreciated that the complexity of abuse and the various ways in which it impacts a person was acknowledged:

*“(There was) recognition that abuse is complicated and often crosses various categories.” Helen, (survey response).*

There was a recognition from those that provided qualitative responses that some of the Truth Project processes helped them feel someone had considered how a survivor might feel in that position. One example of this was that they did not need to repeat their story to various people if they did not want to, which was a key aim of the VSCP when they co-designed the model:

*“The fact that I didn't have to keep repeating who I was, where I came from, and what happened to me helped me to know that I was an individual. I explained things once, and I didn't have to go over it again with another person, it was very helpful and I felt I was a person and not just another victim.” Andrea, (interview).*

However, whilst many felt the Truth Project was able to identify and meet their individual needs, this was clearly within a structure which others appeared to feel was too standardized:

*“They’re coming at it from the angle that they don’t really know what anybody’s going to say or what situation anybody’s in so they have to have a vanilla approach to everybody.” John, (interview).*

### Trustworthiness in the truth project

In relation to the principle of trust, 84.9% (*n* = 56) of participants reported finding the Truth Project trustworthy, while 6% (*n* = 4) said the Inquiry did not act in a trustworthy way and 9.1% (*n* = 6) did not indicate either way.

There was a recognition that trust must be earnt and that the Inquiry did earn this through the way in which they interacted with participants:

*“I feel trust is gained and the support before, during and after made me feel at ease and I trusted the many amazing staff members throughout.” Cathy, (survey response).*

In terms of earning trust, there were many comments about how this was achieved: 6% (*n* = 4) spoke about how confidentiality was maintained within the session and around the environment, as well as how information was handled; while 7.6% (*n* = 5) highlighted the clear communication (through media advertisements or in communication with the inquiry), and the predictability of the process:

*“There was an integrity between what I was told I could expect at the interview, and what actually happened at the interview. It all matched up and really helped me to feel safe - that this was a process with people I could trust.” Andrea, (interview).*

### Feeling empowered and having choice and control

Regarding the question about feeling empowered by the Truth Project, 78.8% (*n* = 52) of participants reported feeling empowered to make decisions when engaging with the Truth Project, 10.6% (*n* = 7) did not, and 10.6% (*n* = 7) did not indicate either way.

Participants highlighted the need to feel heard as being a part of facilitating a sense of empowerment:

*“Trauma survivors want to feel that someone has seen them and is holding them in their strength not as seeing them as fragile or incapable … we often get the message from people in our lives externally that something is wrong with us, we are too emotional or too fragile but we also believe this about ourselves … The truth project did that, held me in my strength.” Diane, (survey response).*

When exploring the extent to which participants felt empowered by the Truth Project, there was a consistent theme around choice and control being important:

*“I had choices about, you know, the times, dates and the gender that I talked to, yes I had those choices.” Vicky, (in interview).*

Individuals appeared to view these principles as important because they were in contrast to the lack of choice or control in their experiences of CSA:

*“I felt like I was in control of what was going on and that my input and that I was important. This felt like the polar opposite of my experience of abuse, where I was not in control, and I was insignificant and did not matter.” Amelia, (survey response).*

One participant described in detail how they experienced each stage of their Truth Project journey and afterwards as empowering:

*“I read the IICSA website a thousand times before I actually summoned up the courage to make contact … once I pressed the on-line button, I felt empowered. … The fact that I could have dedicated time to explain what had happened to me was empowering … Although my contribution was over three years ago, handing over the baton to the inquiry team was so empowering and has considerably helped me with my journey of healing … I feel that if they had not agreed to be on the panel to do their work, my story would have been buried in a secret, unspoken black hole. Sharing my story with a panel member is something I shall never forget - it was so empowering.” Andrea, (interview).*

For others however there were mixed feelings because they felt organizations and agencies that they were referred to after the Truth Project, undid or compromised the feeling of empowerment they had built:

*“After submitting my written statement and feeling like it was important to be heard, I didn’t feel empowered by the police.” Abigail, (survey response).*

### Feeling safe

In exploring the principle of safety, 68% (*n* = 49) reported feeling safe all of the time when engaging with the Truth Project and 28.8% (*n* = 19) reported feeling safe “most of the time,” while 3% (*n* = 2) reported that they felt safe “none of the time.”

Safety, for one individual, was considered both as emotional safety and physical safety:

*“I felt very safe emotionally and physically throughout.” Christina, (in interview).*

A total of, 24.2% (*n* = 16) of participants identified specific actions that they felt the Truth Project was taking to promote safety:

*“The care, empathy, body language, language, all made me feel safe.” Sheila (survey response).*

Some participants, 4.5% (*n* = 3), described elements of the venue that increased their feelings of safety, such as the location of the session or the layout of the room:

*“I loved the huge room I was interviewed in. It was like a hall. So much space made me feel safe.” Andrea, (interview).*

Others considered this in terms of the boundaries and the support offer that was available to them.

*“I always felt safe in the knowledge I could stop anytime and that everything was confidential and there would be support afterwards should I need it.” Bryony, (survey response).*

Safety was also said to be impacted by external factors and concern about what would happen with the information they shared:

*“From the time I made contact I felt safe, my only concern was the police contacting me because I knew they had to report it and this was the really big reason that put me off.” Barbara, (survey response).*

Responses indicated that safety was, to a large extent, based on interactions with Truth Project staff. This included all staff, such as receptionists smiling and being welcoming, interactions on the phone, support given as well as staff in session.

*“I felt that I was not judged, and that those around me were not shocked by anything disclosed therefore I felt safe from disclosing the information.” Naomi, (survey response).*

### Being believed

A central part of the Truth Project is that victim and survivor’s accounts are not questioned or challenged and the information they provide is not verified or tested, 13.6% (*n* = 9) of participants discussed this theme.

Five participants indicated that this sense of belief facilitated engagement and shaped their experience, putting victims and survivors in a better mental space to share:

*“The idea of the name ‘the Truth Project’ automatically sends the signal that whatever you say will be taken as the truth. The truth until proven otherwise.” Vanessa (interview).*

A further 6% (*n* = 4) noted that this aided disclosure as there was no pressure to have to substantiate what they were sharing:

*“Every piece of information was accepted at face value, they appear to trust implicitly what I disclosed.” Shana (survey response).*

This sense of being believed helped shift how their experience was understood and framed, which helped the healing process:

*“The abuse I suffered as a child was truly acknowledged in the session and by the support I was given before and after it. It had never been acknowledged as serious before even by my parents and I found my experience with the Truth Project immensely healing as a result.” John (survey response).*

*“It had always been something that I had tried to minimize, ignore and even deny it although I believe that it did cause me harm. To have the reality of that harm acknowledged has made a huge difference to me.” Amelia (survey response).*

### Long term consequences and support considerations

The majority of participants indicated that they considered the support during engagement with the Truth Project was helpful and appropriate. However, 16.7% (*n* = 11) did report that they needed longer term support following their Truth Project session:

*“I feel that the inquiry is trauma informed but I wonder if it is aware of the long-term psychological damage that can be caused for people who open up for the first time ever and then leave the inquiry without any follow up support.” Rachel (survey response).*

Without this additional support being available, some participants reported still experiencing negative consequences of having engaged with the Truth Project, at the point of their responses:

*“More support needed for survivor's. My life has spiraled out of control and I have had another breakdown. I lost my job and am on the brink of losing my marriage. Having to fight another battle is a struggle, I'm doing it alone again …” Lauren (Interview).*

Of the 6% (*n* = 4) who reported to still be struggling with the impact of sharing their experience at the time of responding to the survey, two of them had experienced their session between 1 and 3 years prior to their engagement in this project. A further two were more than 3 years post session:

*“I was very overwhelmed with the whole experience and apart from the support up until 2 weeks after I've been unable to get support since. I’m struggling more than ever and do regret doing the truth project now knowing how much it has affected me.” Natalie (interview).*

Whilst the majority of participants felt that their engagement with the Truth Project was trauma informed, the process of having their information shared with the police and their subsequent interactions with police, were reported as not:

*“The only negative experience I had was with the Police contact after my Truth session … I did find that upsetting and contrary to the rest of my experience.” Amelia (interview).*

## Discussion

Overall, these findings suggest that the hybrid survivor co-designed TIA employed by the Truth Project was well received by the majority of participants and facilitated their engagement. The data around the extent to which the Truth Project process was retraumatizing and to what extent the TIA was able to fully mitigate this risk was more mixed. Over a third of participants identified the Truth Project process as at times retraumatizing. The qualitative data suggests that for some participants, talking about CSA carries an inevitable element of reexperiencing that is difficult but manageable. For a small minority however, what engagement with the Truth Project brought up for them was retraumatizing in a way that had long lasting effects on their mental health. A final theme that was not directly asked about but which emerged in the qualitative data analysis was the importance of being believed in their contact with the Truth Project. Belief was linked to longer term healing from the impacts of CSA and previous societal responses to participants.

### Historical institutional abuse inquiry scholarship and clinical implications

HIA Inquiries have become a new area of scholarship, with a particular focus on how participation is experienced by victims and survivors and to what extent their needs are taken into account (14, 17). This study adds to that literature and in evaluating the impacts of using a TIA, demonstrates that for many survivors support needs can be addressed via therapeutic means. However there are important caveats to this finding, both in relation to the minority of participants who identified the process as harmful, and also in the scope of what this study investigated in comparison to the broader literature. Based on these findings, while trust was established for the majority, choice and control were offered, and a number of participants described feeling empowered, the levels of retraumatization are of concern. Similarly to Hamber and Lundy’s 2020 study in Northern Ireland (17), it would appear that while participation in these forms of HIA Inquiries confer meaningful benefits for victims, these are not without risk, and this needs to be explicitly communicated prior to participation. A key difference between this study and Hamber and Lundy (17), is that the scope of this study was focused on victim experience of the process and did not investigate wider justice needs.

While this study has focused on individual experience, there is support for victim needs being considered in a wider social and political context, with attention focused on a range of justice outcomes (16). This study also supports participation Inquiries as one way to promote healing for survivors of CSA, adding evidence for an approach that validates survivor testimony, and places dignity, as an antidote to shame, at the heart of participation (18, 47). The theme of being believed and having accounts validated, supports the benefits of participation in Inquiries as citizens in addition to survivors, and lends further evidence to these processes as forms of therapeutic politics that can lead to personal healing and social change (19). Based on the findings of this study, it appears that providing survivors with an opportunity to share their experiences offers a form of epistemic justice to repair previous injustices. This took the form of both testimonial and hermeneutical justice. The Truth Project offered a testimonial justice by addressing a previous “credibility deficit” (20) whereby the survivor has had their account invalidated as a result of being a child, evoking prejudice in the listener because of prejudice, or because of the relative credibility of the abuser who denies the allegation. A key form of hermeneutical justice offered is the public visibility of the Truth Project enabling survivors to feel less alone in their abuse, and to develop a new, destigmatised way of talking about non-recent CSA that was previously unavailable.

From a clinical perspective, several participants picked up on the detailed environmental context (room furnishings, refreshments and use of space), and interpersonal qualities of Truth Project staff as key to their overall experience. Through consultation with the VSCP, it became clear that a lot of this attention to detail arose through their “walk through” of the Truth Project process and their authorship of aspects of the model. While the TIA literature in general advocates survivor involvement in service design (33) this can often be backgrounded (48). Based upon these findings, survivor involvement in the design of TIAs are central to the translation of the components into a “felt” sense for Truth Project participants, particularly around creating non-clinical, welcoming environments. The VSCP can be seen to have helped IICSA develop what Fricker has described as a “testimonial sensibility” whereby they were able to take a “critical openness” to listening to survivor accounts. What is of importance is that this sensibility is not rule bound, or at least should not be when fully realized (20). Rather it relies on “the educated improvisations of a moral perceptual sensitivity [(20), p. 73], somewhat akin to other forms of improvisation in artistic endeavors. Given the IICSA staff group were largely made up of civil servants, there is likely to have been challenges for them and the VSCP in encouraging the development of an improvised approach to the development of an organizational ethical consciousness. Perhaps the TIA “rules” were necessary in supporting the imitation of this form of virtue, but there is evidence in the data that some survivors experienced the TIA being integrated in a seamless way, that suggests a less rigid approach. The work the VSCP did as the “in house” survivors, and keepers of knowledge of the dangers of epistemic injustices appears to have been key in creating a milieu where communicating belief was a central task.

This study goes some way to challenge a widely held belief that people can only talk about experiences of CSA in long term therapeutic interventions, and that anything else risks leading to destabilization (49). The majority of the current sample reported positive experiences from participation. The study supports the positive benefits of talking about CSA, in order to challenge stigma and silencing in services, and also wider society (24).

A final clinical implication for future Inquiries is the lack of longer-term support. While choice and control was raised as a positive component by some participants, engagement with the Truth Project was time-limited. This was raised by some of the participants who experienced some reemergence of post traumatic symptoms after their engagement. It may be that future HIA Inquiries can build in flexibility about what longer term support is available, to give participants more choice over the care they receive and to respond to people who have negative responses to participation.

### Trauma informed approaches

The findings of this study, around the use of TIAs to support adult victims and survivors of child sexual abuse, fits broadly into the wider evidence base for TIAs. Similarly to a recently conducted systematic review (39) there is evidence from this study that the emphasis on staff training, interpersonal skills, environmental adaptation, and responding sensitively to trauma disclosure were all positively connotated by participants. TIA as a service model had meaningful connotations for many participants and they recognized the importance of a TIA implementation as highly relevant given the population and subject matter. While there is risk of branding over substance in TIA implementation (48) if properly operationalized, TIA as a service design model and philosophical orientation may be adaptable for other non-clinical service settings trauma victims and survivors engage with, such as justice and welfare systems. The TIA staff training implemented by the Truth Project was noticed by several participants and demonstrates that non-clinical staff can be trained in a TIA model and adapt their communication style to take account of trauma. The importance of survivor participation in the TIA development and in staff training was critical in the Truth Project.

Another finding that is pertinent for TIA research is the reported impact that it has when several participants in this study engaged in services such as some police services as a result of their contact with the Truth Project. This is a considerable challenge for service settings with significant safeguarding responsibilities, where the survivor has contracted to work in a TIA service setting but is then referred to external agencies who respond to trauma differently. This study would suggest the importance of making clear to victims and survivors at the contracting stage that external agencies may respond differently to trauma responses.

A final finding pertains to an additional theme, the importance of being believed. This has implications for the links between TIAs and epistemic justice, showing a potential gap in the constructs TIAs incorporate. Fricker suggests, “epistemic trust incorporates ethical trust … seeing a speaker in epistemic color entails seeing them in moral color” [(20), p. 76]. From this perspective not only is belief communicating the epistemic worth of a speaker, it also carries moral weight by recognizing their sincerity. There is no formal recognition of the need to believe trauma survivors in the TIA literature, it is incorporated in other constructs such as understanding the person through a trauma lens (33), but based on this study it could be explicitly included. The communication of belief could also form one part of the dignity conferring processes that are seen as an antidote to the inherent shame of sexual abuse (47).

### The truth project and epistemic justice

Considering the research findings in light of the epistemic injustice literature, there are a number of themes of interest. As discussed above, the importance of being believed by the Truth Project was so central to several participants, that they brought it up unprompted. The Truth Project’s most obvious achievement is through what is communicated by its name and the ethos of the methodology that arises from it, survivors were believed and not questioned. This granted them what Fricker describes as epistemic relational equality, to compensate for their historic blocking rom making an epistemic contribution (22). It can be argued based on this approach, that the epistemic inequality survivors of CSA have historically faced are linked to other forms of social and economic inequality. The Truth project’s own data found a high proportion of survivors described the impacts of CSA on their educational and vocational development, also unsurprising given that outside the home the most frequently cited source of abuse occurred in educational settings (Truth Project dashboard). It is reasonable to wonder if the lack of epistemic equality arising from their CSA experiences are compounded by other, intersectional forms of epistemic injustices based on other aspects of their identity such as gender, mental health status, social class and social capital. One can see the CSA as an original injury that impacts development in ways that increasingly marginalize the person’s epistemic worth, making it more challenging for them to be taken seriously as reliable witnesses to their own experience and simultaneously excluding them from a hermeneutical justice whereby they could make meaning of their CSA experiences through engagement with other survivors. One the of most consistently reported impacts of the Truth Project, both in this study and in other forms of feedback to IICSA, was how meaningful it was for people to realize they were not alone in what they had experienced.

It may be that the Truth Project can offer ongoing epistemic legitimacy to CSA by virtue of the scale of participation, however historical analysis would suggest that CSA occupies a paradoxical space in the public imagination, by turns hyper-visible and prone to outraged reactions, followed by periods of denial and disbelief (24, 50). It is likely to fall to activists in general, and survivors in particular, to continue to remind the public and public bodies about the scale of CSA and its impacts which the Truth Project has uncovered. This work carries with it complex demands of survivors, including the emotional labor and risk of retraumatization described in this study (13). A question it raises is that while it is laudable that the Truth Project has advanced the epistemic credibility of CSA survivors by offering a hermeneutical language whereby non-recent CSA can be spoken about by survivors in credible ways, it is another question entirely about whether they should be expected to do so. A key question for clinicians and researchers working in the field of non-recent CSA is what forms of advocacy are needed from them to support CSA survivors without coopting their epistemic claims and translating them into professionalized discourses (51).

This study also links to other scholarship in the area of epistemic justice and mental health. One recent study considered the legitimation of user knowledge in mental health services through a participatory research methodology (23). While they found evidence of support for user involvement in knowledge construction, there were limits to the reach of these forms of knowledge. Similarly in this study, while the Truth Project offered a platform of belief and validation of survivor testimony, it did not necessarily transfer to the epistemic demands of other settings such as the criminal justice system. So while the testimony was accepted as reliable within the confines of the Truth Project, it was not considered to have the same epistemic status as evidence provided at a Public Hearing, something which was similar to other Inquiries (17). This links to an important finding of this study that the TIA operated by the Truth Project was not replicated in adjacent organizations such as the police, that survivors were referred onto. This raises the possibility that the contingent nature of the epistemic legitimacy offered by the Truth Project could set survivors up to fail when they take their knowledge claims into other settings, with a different politics of knowing. Wider culture change to match the development of epistemic justice in one area of public life, needs to be matched by partner agencies, a finding picked up by another study which looked at staff attitudes to increased epistemic agency amongst inpatient adolescent service users (52).

One contribution this study has made to the epistemic injustice literature, is to operationalize some of the philosophical concepts in a way that can be applied. While Fricker suggests it is problematic to turn a testimonial sensibility into a form that makes moral knowledge codifiable, she does point out that rules can offer guidance for someone “en route” to full virute, while not being a substitute for it [(20), p. 73]. Based on the findings in this study it was possible to at least develop a set of rules for engaging ethically with CSA survivors through the TIA, and, crucially, the inclusion of the VSCP in developing a survivor oriented TIA.

The current study was limited in a number of respects. Most significantly was the independence of the evaluation, both perceived and actual. While none of the researchers were involved in the design and initial implementation of the original TIA in the Truth Project, all were involved in its later implementation and were Inquiry employees during the evaluation. From the participant perspective, they were recruited via an IICSA group, the VSF, and so will have been aware that it was the Inquiry itself seeking feedback, potentially skewing what was reported. However the presence in the sample of participants who had more difficult experiences with the Truth Project suggests there were a range of views reported.

## Conclusion

This study was a mixed-methods survey based evaluation of a large-scale HIA Inquiry’s engagement with adult victims and survivors of CSA. It focused on the implementation of a survivor co-designed TIA that was designed to address victim needs when they shared their experience with the Inquiry in a private capacity. The findings suggest that most participants in the study sample found the TIA addressed their needs and while there was some evidence of longer-term detrimental impacts, this was in a small minority of cases. While more focused research on outcomes needs to be undertaken, there is some support for the use of survivor co-designed TIAs in the support of victims and survivors of child abuse engaging with HIAs. An important component of the TIA was the survivor amendments to the model, which focused on aspects that may have been missed by top-down implementation. The wider significance of the Truth Project is that it challenges long held beliefs about the value of talking about CSA in a safe, supportive environment where belief, validation and dignity are prioritized. Fricker’s work largely draws on exemplars of epistemic injustices and their antithesis from literature to elucidate her arguments (20). Findings from this study exemplify the process of moving from a position of experiencing prejudice and isolation as a knower, to feeling included in a wider network of meaning making in the field of CSA, and by virtue of participation achieving forms of testimonial and hermeneutical justice. It is suggested that future studies could operationalize Fricker’s model through engagement with other groups who also face Epistemic Injustices.

## Data availability statement

The raw data supporting the conclusions of this article will be made available by the authors, without undue reservation.

## Ethics statement

The studies involving human participants were reviewed and approved by IICSA independent ethics committee. The patients/participants provided their written informed consent to participate in this study.

## Author contributions

CB: project manager, collected data, analysed data, and contributed to write up. SF: collected data, analysed data, and contributed to write up. RE: project leadership and liaised with organization. SQ: collected and analysed data. DT: research supervisor and led on write up. All authors contributed to the article and approved the submitted version.

## Funding

Funding coming via Home Office/IICSA due to research being public funded and so OA a requisite.

## Conflict of interest

The authors declare that the research was conducted in the absence of any commercial or financial relationships that could be construed as a potential conflict of interest.

## Publisher’s note

All claims expressed in this article are solely those of the authors and do not necessarily represent those of their affiliated organizations, or those of the publisher, the editors and the reviewers. Any product that may be evaluated in this article, or claim that may be made by its manufacturer, is not guaranteed or endorsed by the publisher.

## Footnotes

^4^It was noted that there was a higher proportion of female respondents to the mixed-methods survey and a slightly lower proportion of white British participants, resulting in a more ethnically diverse sample when compared to Truth Project data (https://www.iicsa.org.uk/key-documents/26714/view/truth-project-dashboard-august-2021.pdf).
